# Dr. Mütter

**Published:** 1859-05

**Authors:** 


### NECROLOGICAL NOTICES.

DEATH OF DR. MUTTER.—Within the year which closed with the end of March, 1859, two members of the most successful medical faculty which this country has seen, have passed from the scene of their labors and triumphs to a world where men toil not, nor spin, and where the ambitions of this earth live no longer. Of one of these friends, it scarcely becomes the writer to speak; kind and tender hearts, friends won by long service, and a profession served and elevated by earnest and happily-directed industry, have spoken his eulogy in many ways, and in terms which are deeply felt by those who were most near to him in blood and friendship. Such ties united him to the subject of this memoir, that it is impossible to do otherwise than connect them in thought, now that death, which severed their union, has come with thoughtful charity to reunite them again. Both were Virginians, of a school almost extinct; both were trained to the courtly manners of an elder day; both were alike remarkable for professional skill and for a hospitality as warm as the hearts which prompted it.

The Rev. Frederick Ogilby, of New York, a friend of Dr. Mütter's, as well as of his colleague, has expressed in a few sentiments, and with graceful feeling, the ties which united the two Professors and the points in which they resembled one another. He says:—

"On opposite corners of Walnut and Eleventh Streets lived two Virginians; not such as Thackeray imagines, but real native-born, actual Virginia gentlemen. They were both physicians, Professors in Jefferson College, both distinguished for the highest attainments in their profession, and both true men, whose hearts and doors were always open. In their lives they were united, and in their death they are not divided. Only a few short months have intervened between the deaths of Dr. J. K. Mitchell and Dr. Thomas D. Mütter. One familiar with Philadelphia for the last few years must feel how much its society has lost in the death of two such men. It is happy now to think, at least the writer of this rejoices in such a thought over the graves of beloved friends, that to their earthly distinction they added the gifts and qualities of true-hearted Christians."

Dr. Thomas Dent Mütter was born in Eppes Street, Richmond, Virginia, on the 9th of April, 1811. He was descended from Thomas Mütter, a merchant of Glasgow, in Scotland, who emigrated to North Carolina before the Revolution, and became connected by marriage with the family of Moore, two of whose members were distinguished for their gallantry in the battle of King's Mountain and other actions. Mr. John Mütter, the father of the subject of this sketch, removed to Richmond in early life, and was well known in that city as a successful merchant, and not less for the exercise of a hospitality such as was remarkable, even in a State proverbial for its liberal greetings and luxurious ease. How well Dr. Mütter himself sustained the character of his Virginia home, is known to most of us, and will be long remembered by those also who have shared the generous hospitalities of his heart and house.

Dr. Mütter's mother was Miss Gillies, of Alexandria, a half-sister of the late General Walker Armistead, of the United States Army. Through this lady, he was also connected with Gillies, the historian of Greece, and with the well-known families of Carter, Lee, and Dulaney. Professor Mütter had the misfortune to lose both his parents before he attained his ninth year. Thus left alone in the world, he fell to the kind charge of his guardian and kinsman, Colonel Robert Carter, of Sabine Hall, where he spent his boyhood. He received his classical education at Hampden and Sydney College, and afterwards spent a short time at Yale, where he intended to pursue still further his preparatory studies, an intention which was frustrated by an attack of hemorrhage from the lungs, which induced him to give up his plan of temporary residence in a far northern climate. He accordingly removed to Alexandria, where he began to study medicine in the office of Dr. Simms, of that city. His subsequent studies were pursued in the University of Pennsylvania, under the immediate tuition of Dr. Samuel Jackson, still the honored Professor in that institution. Dr. Mütter graduated in 1831, at the early age of twenty years, and in the spring of the same year sailed for Europe, and went to Paris, where he devoted himself to surgical pursuits, and especially to the clinics and lectures of Dupuytren.

After residing in Europe about one year, Dr. Mütter returned to this city, where he began to practice his profession, and to instruct students, with whom he soon became deservedly popular. He was at this time one of the first to initiate the practice of recapitulating, by a series of questions, the lectures to which his pupils had listened during the week. This mode of teaching has since held a recognized place among us, and is certainly of value so long as it does not degenerate into a mere system of verbal drill.

Like most of our best physicians, Dr. Mütter obtained his first independent experience in the Philadelphia Dispensary, and not long after he began to teach, received an appointment in the summer school, founded by Dr. Chapman. Here he displayed the remarkable powers as a teacher which have since become more widely known. Meanwhile, a large and increasing surgical and general practice, combined with his growing fame as a teacher, rendered him a prominent candidate for any vacant chair of surgery or anatomy. The reorganization of the Jefferson Medical College, in 1841, afforded the wished-for opportunity, and in company with his friend, Dr. Mitchell, the subject of this notice entered the ranks of the new faculty. A triumph scarcely anticipated by the most hopeful of those who had entered into this new enterprise awaited its future. To this result the young Professor of Surgery largely contributed; and when the new faculty planned and carried out their system of clinical lectures, Dr. Mütter's skill as a surgical teacher and a dexterous operator still further added to his reputation, and to that of the College. To increase the interest and usefulness of his clinic, no sacrifice was too great—no trouble to be counted. The excellent results of this method of practical teaching, was also aided by the perfect understanding which existed between himself and the Professor of Anatomy, Dr. Pancoast. At every important operation they assisted each other, and frequently brought to the public clinic their private patients.

Dr. Mütter's merits as a teacher of surgery, were of a rare order. We have heard as good clinical lecturers, but we have heard no mere didactic teacher of surgery whose lectures surpassed those of Dr. Mütter in all the essentials of teaching.

From the ample resources of his museum the arena of his lecture theatre was filled with illustrations, casts, morbid specimens, etc. The lecturer used no notes, save a page of his syllabus. His manner was most impressive; few lecturers had better listeners. His small and graceful frame, and the quiet ease of his social chat, scarcely prepared one for the full tones and emphatic manner in which he laid down the principles of his art.

Dr. Mütter's reputation as an operative surgeon, was deservedly high. A monograph upon club-foot, and several papers recording most daring plastic operations for the cure of the deformities resulting from burns, indicate the direction which his surgical reputation took. He had the best qualities of a good operator, and although during later years his declining health indisposed him to attempt severe operations, earlier in his career no case was too formidable. Under an appearance of excessive—almost nervous—anxiety, which was largely due to ill-health, he concealed a real coolness and confidence which no surgical emergency disturbed. Again and again, the writer has seen him suddenly tested by unlooked-for contingencies which, neither in public nor private, exhausted his resources or affected his powers. As a surgical physician, if such a term be allowable, Dr. Mütter was remarkable for the singular skill which he exhibited in the diagnosis of surgical cases. He possessed, most amply, that union of qualities which constitutes surgical tact, while no man was more frank in his prognosis, and, certainly, none more careful and untiring in the after-treatment of those who had undergone the prompt remedy of the knife. The daring which inspired his operative practice, was no less visible in the readiness with which he accepted and made use of the novelties of the day, a peculiarity which did not lessen, as too often happens, with advancing years.

Three years ago, Dr. Mütter's failing health convinced him that he was no longer able to fulfill the duties of his chair, and, although his colleagues were willing to afford him any indulgence, he was indisposed to continue in a position which he could not fill with entire satisfaction to his own sense of right. He, therefore, resigned his office, and was, shortly afterwards, appointed Emeritus Professor of Surgery in the College whose fortunes he had so eminently aided by his labors.

In the ensuing autumn, Dr. Mütter sailed for Europe, in search of health. Before he left this country, he entered into negotiations with the College of Physicians of Philadelphia, intending to give to that learned body his valuable museum, and to endow it with 30,000 dollars, so soon as its Fellows should have succeeded in erecting a suitable building to accommodate the collection. His rapidly failing health, and the delay necessarily attendant upon so important a matter of business, induced Dr. Mütter to postpone the conclusion of his negotiation with the College, and after providing in his will for the ultimate fulfillment of his intentions, he sailed for England.

A protracted residence abroad brought no relief to the malady whose only remedy was to be its own last and fatal attack. Weary with the endless torture of disease, he returned to this city in the autumn of 1858, when he hastened to fulfill his promise to the College of Physicians, as though conscious how little time was left him for the affairs of this world. Upon the signature of the papers necessary to complete his contract with the College, Dr. Mütter left this city again, in search of the health which came no more, and turned his steps toward the South, where he spent the last winter between Savannah and Charleston. In this latter city, his constitutional malady rapidly increased upon him, and on the 16th of March, he finally passed away from the kindness and generous sympathies which his character and social graces had gathered around him in a land of strangers.

This kind and Christian heart, this generous and accomplished physician, has at length found the sad relief which his own art denied him here. Among those to whom he gave that ease from suffering in its manifold varieties, which he sought in vain, there will be many to regret his loss; and in the ranks of the profession which gave him all its honors, his remembrance will be held in regard, not less for the qualities which earned success than for the generous spirit with which he returned to his mother art the riches she had given.

One word more. From boyhood to the day of his death, the subject of this sketch was the victim of hereditary gout, which preyed endlessly upon a frame inconceivably sensitive to pain. In this round of torture there were change and variety of affliction, but of late years, scarce any interval of entire relief. Into the estimate of his labors, and of his character, this element should fully enter.

With this brief record of a weary and burdened, but fruitful life, we take leave of one whose kindly sympathies and ready courtesy will long be missed and mourned in the home of his adoption.

